# Isolation and characterization of a N4-like lytic bacteriophage infecting *Vibrio splendidus*, a pathogen of fish and bivalves

**DOI:** 10.1371/journal.pone.0190083

**Published:** 2017-12-28

**Authors:** Pantelis Katharios, Panos G. Kalatzis, Constantina Kokkari, Elena Sarropoulou, Mathias Middelboe

**Affiliations:** 1 Institute of Marine Biology, Biotechnology and Aquaculture, Hellenic Centre for Marine Research, Crete, Greece; 2 Marine Biological Section, University of Copenhagen, Helsingør, Denmark; Universidad Miguel Hernandez de Elche, SPAIN

## Abstract

A novel virulent bacteriophage, vB_VspP_pVa5, infecting a strain of *Vibrio splendidus* was isolated from a sea-cage aquaculture farm in Greece, and characterized using microbiological methods and genomic analysis. Bacteriophage vB_VspP_pVa5 is a N4-like podovirus with an icosahedral head measuring 85 nm in length and a short non-contractile tail. The phage had a narrow host range infecting only the bacterial host, a latent period of 30 min and a burst size of 24 virions per infected bacterium. Its genome size was 78,145 bp and genomic analysis identified 107 densely-packed genes, 40 of which could be annotated. In addition to the very large virion encapsulated DNA-dependent RNA polymerase which is the signature of the N4-like genus, an interesting feature of the novel phage is the presence of a self-splicing group I intron in the thymidylate synthase gene. A tRNA^Stop^ interrupted by a ~2.5kb open reading frame–containing area was also identified. The absence of genes related to lysogeny along with the high efficacy observed during *in vitro* cell lysis trials, indicate that the vB_VspP_pVa5 is a potential candidate component in a bacteriophage cocktail suitable for the biological control of *V*. *splendidus* in aquaculture.

## Introduction

*Vibrio splendidus* is a ubiquitous inhabitant of marine and brackish water with prodigious genotypic diversity that plays a major ecological part in the oceanic and coastal environments [1,2]. It has been associated with severe epizootics in many farmed aquatic animals including fishes [3–5], crustaceans [6], bivalves and echinoderms [7]. In bivalve aquaculture, *V*. *splendidus* is considered to be one of the most important bacterial pathogens [8–11] responsible for severe financial losses, while in fish culture it has been reported to cause significant mortalities mostly in turbot larvae [3,12]. Skin Ulceration Syndrome (SUS) caused by *V*. *splendidus* [7] is an important threat for the viability and profitability of the rapidly expanding industry of holothurian culture in China and Southeast Asia [13]. In fish and invertebrate larviculture where the use of antibiotics has specific drawbacks and therefore is not applicable, control of vibriosis is extremely challenging. In these cases, bacterial control is based on the enhancement of the innate immune system of the farmed animals [14] or on the use of probiotic bacteria [7,15].

Phages have been considered as a promising alternative to antibiotics since they present several benefits over chemotherapy for microbial control. These include their high host specificity indicating that they are harmless to the natural microbiota and their autonomous transfer between animals following initial administration [16]. Phage therapy could be an ideal option for microbial control in the fragile environments of the fish and invertebrate hatcheries [17].

A few phages infecting *V*. *splendidus* have previously been isolated and phage therapy against *V*. *splendidus* in sea cucumber (*Apostichopus japonicus*), hatcheries has recently produced encouraging results leading to increased survival of the phage- treated populations. However, the phages used in that study, PVS-1, PVS-2 and PVS-3 were only morphologically characterized [18]. Additionally, although the genome sequences of three more phage isolates infecting *V*. *splendidus* (Helene, Henriette and Martha) are available in GenBank, as yet no publications about them have been released.

Here we report the isolation and characterization of vB_VspP_pVa5 (pVa5), a lytic N4-like podovirus which infects a *V*. *splendidus* strain. To our knowledge, this is the first *V*. *splendidus* phage characterized both morphologically and genetically.

## Materials and methods

### Bacterial strains

The bacterial strain used as a host (VaAn) was isolated from mussels of a bio-fouled fish cage in Greece during a vibriosis incidence. The bacterial strain has previously been fully sequenced (Accession number: PRJNA349813) and identified as *Vibrio splendidus* by multilocus sequence analysis (MLSA) [19]. It is a moderate virulent strain able to cause mortality to fish as assessed in *in vivo* studies using cod, turbot and halibut larvae [19] and is resistant to sulfadiazine/trimethroprim and oxytetracycline. In addition to the host, ten *Vibrio splendidus* strains and four clinical *Vibrios* associated with fish diseases from HCMR’s collection were used in order to assess the host range of the phage. The strains used in this assay are presented in Table 1. Bacterial strains were maintained in microbeads (MicroBank) at -80°C and grown in sea water supplemented with 1% tryptone and 0.5% yeast extract at 25°C. Host range was assessed using the agar-overlay method [20].

**Table 1:** Host range of pVa5.

#	Strain	Bacterial species	Origin	Infectivity
**1**	VaAn	*V*. *splendidus*	Greece	+
**2**	3D3-3/pop17	*V*. *splendidus*	France	-
**3**	4D6-8/pop19	*V*. *splendidus*	France	-
**4**	3F1-17/pop25	*V*. *splendidus*	France	-
**5**	3F1-22/pop21	*V*. *splendidus*	France	-
**6**	3F1-45/pop20	*V*. *splendidus*	France	-
**7**	3H2-4/pop24	*V*. *splendidus*	France	-
**8**	3Z-15/pop23	*V*. *splendidus*	France	-
**9**	3Z-28/pop22	*V*. *splendidus*	France	-
**10**	3Z-31/pop16	*V*. *splendidus*	France	-
**11**	3Z-39/pop18	*V*. *splendidus*	France	-
**12**	DSMZ 2171	*V*. *alginolyticus*	Japan	-
**13**	DSMZ 19623	*V*. *harveyi*	USA	-
**14**	ATCC 19264	*V*. *anguillarum*	Sweden	-
**15**	ATCC 33509	*V*. *ordalii*	USA	-

### Phage isolation

The phage was isolated from sea water near a cage aquaculture farm in central Greece in January 2015. Water samples (1 L) were supplemented with 1% tryptone (Difco) and 0.5% yeast extract (Difco), inoculated with VaAn and incubated at 25°C for 24h. Following filtration through 0.22 μm filters, 20 μL aliquots were tested for clearing zones on bacterial lawns of the host strain. Single phage plaques were detected following serial dilutions. Isolated plaques were picked and purified by re-plating five times to ensure clonal phage stock. The purified phage was propagated in high titer (10^10^ pfu mL^-1^) and stored at 4°C. The phage and its bacterial host VaAn were deposited in the open collection of the Leibniz Institute—Deutsche Sammlung von Mikroorganismen und Zellkulturen (DSMZ) under the accession numbers DSM 104622 and DSM 104620, respectively.

### Phage morphology

Phage preparation in high titer (10^10^ pfu mL^-1^) was negatively stained with 4% (w/v) uranyl acetate (pH 7.2) and observed with a JOEL JEM2100 transmission electron microscope (TEM) operated at 60 kV at the Electron Microscopy Laboratory of the University of Crete.

### One-step growth analysis

The latency period and burst size were determined from the one-step growth curve, according to standard methodology. To summarize, 1 mL from a bacterial culture at the exponential phase was centrifuged and washed with saline buffer (0.9% NaCl), re-suspended in 1 ml medium and then infected with the phage at a MOI of 0.01. After 10 minutes’ incubation, the mixture was diluted 25 times in the medium and at this time point and every 10 minutes thereafter, a sample was removed, and serial decimal dilutions were spotted on a previously prepared lawn of the host on agar plates. The number of infective phages in each sample was quantified from the plaques formed after an overnight incubation of the plates [17].

### *In vitro* lysis

An *in vitro* lysis assay was performed in sterile 96-well plates using a TECAN microplate reader (Infinite PRO 200) equipped with temperature control. Specifically, twelve 200-μL wells of the plate were loaded with freshly-prepared cultures of the host organism. The plate was placed in the reader and incubated at 25°C with orbital shaking. Phage was added at three different multiplicities of infection (MOI 1, 10 and 100) in triplicates when the bacterial culture was at the exponential phase. Three wells were not infected and these served as control. The growth curve of the cultures was monitored in real-time over an 8-hour period, and optical density measurements at a wavelength of 560 nm (OD560) were recorded every 10 minutes.

### DNA extraction and sequencing

The bacteriophage’s genomic DNA was extracted according to the phenol-chloroform protocol [21] and stored in sterile Eppendorf tubes in -20°C until sequenced. The genome of the pVa5 bacteriophage was sequenced using the Genome Sequencer GS Junior System (Roche Diagnostic). Shotgun sequencing was performed according to the manufacturer’s instructions using 5 μg of the bacteriophage genomic DNA. Low quality sequences were trimmed off and shotgun reads were assembled using the De novo assembly of the GS Assembler software (Newbler).

### Genome structure determination

The physical structure of the genome was determined by digesting the phage genome with restriction endonucleases. The enzyme ApaI (Thermo Scientific) was used in order to obtain the genomic restriction profile of the phage. The results were visualized on 1% agarose gel and the sizes of the DNA fragments were estimated in comparison with Lambda DNA/Hind III marker (Thermo Scientific).

Additionally, four sets of primers (Eurofins) were manually designed in order to determine by Polymerase Chain Reaction (PCR), whether the phage genome was circularly-permuted (S1 Fig). Two sets of primers specifically targeted two genomic areas which enclosed the first and the last bp of the obtained *de novo* assembled phage genome contig. The other two sets of primers were used as positive controls for the PCR, since they were targeting two random genomic areas of the phage.

### Genomic analysis and phylogeny

Open reading frames (ORFs) were determined using RAST [22,23] and then manually curated using Geneious 9.1.5 based on three different start codons (ATG, GTG and TTG) and putative Shine Dalgarno sequences upstream the coding regions [24]. Genes and coding DNA sequences (CDSs) were predicted using a combined gene model from GeneMarkS [25] and Glimmer3 [26]. The predicted proteins were annotated by BLAST against the NCBI non-redundant database. Additional annotation was subsequently performed using Blast2Go [27] and InterProscan [28]. The presence of tRNAs was examined using both tRNAscan [29] and ARAGORN [30]. The annotated genome sequence for the phage pVa5 was deposited in GenBank database under the accession number KX889068.

Phylogenetic analysis was performed using two genetic markers: the DNA polymerase (DNAP I) and the virion-encapsulated RNA polymerase genes (vRNAP) of 30 available N4-like phages, including pVa5. The selected DNA sequences were aligned by ClustalX with default settings and the phylogenetic tree was constructed using Neighbor-Joining method with 1000 bootstraps through Geneious bioinformatic platform 9.1.5 [24]. The genomes of the N4-like phages which were classified in the same taxonomic group with pVa5 were compared to the novel phage using the progressive Mauve algorithm [31] in Geneious platform 9.1.5, in order to unravel its genomic architecture [24]. The determination of protein structure and RNA families was determined by Phyre^2^ [32] and Rfam [33] online bioinformatic tools, respectively, in those cases where such analyses were necessary. The evaluation of the bacteriophage’s lifestyle was performed through the combination of results obtained by Phyre^2^ and BLAST. The presence of genes, such as integrase, fitness or virulence factors, which might indicate a lysogenic lifestyle for pVa5 was examined.

## Results and discussion

### Morphology, life cycle and phage efficacy

**Fig 1:**
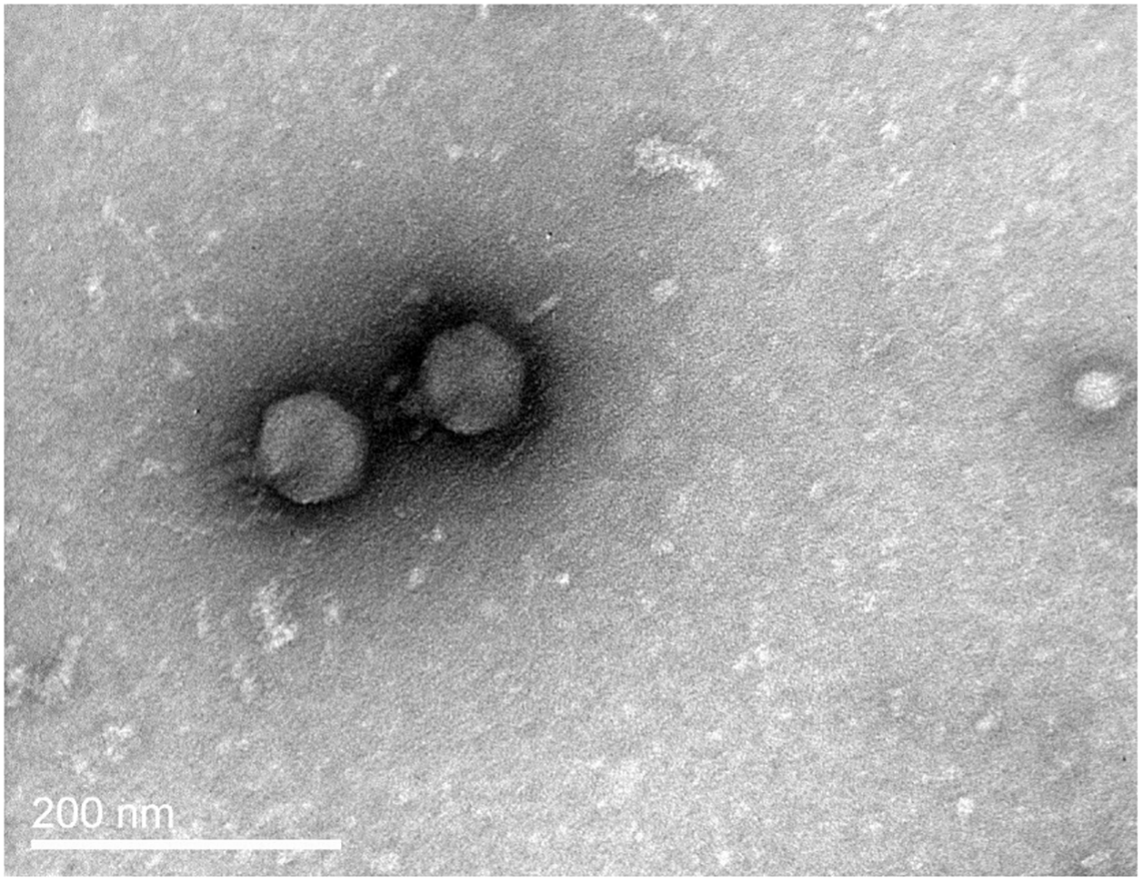
Transmission electron micrograph of *Vibrio splendidus* phage demonstrating a Podoviridae morphology.

The phage produced small (pinhead size) clear plaques on the host lawn. Following TEM observations, it was classified to Podoviridae family. It had an icosahedral head measuring 85 nm in length and a short (25 nm) non-contractile tail (Fig 1). It was designated as vB_VspP_pVa5 according to the suggestions for bacterial viruses’ nomenclature [34], while its abbreviation, pVa5, will be used in the manuscript.

**Fig 2:**
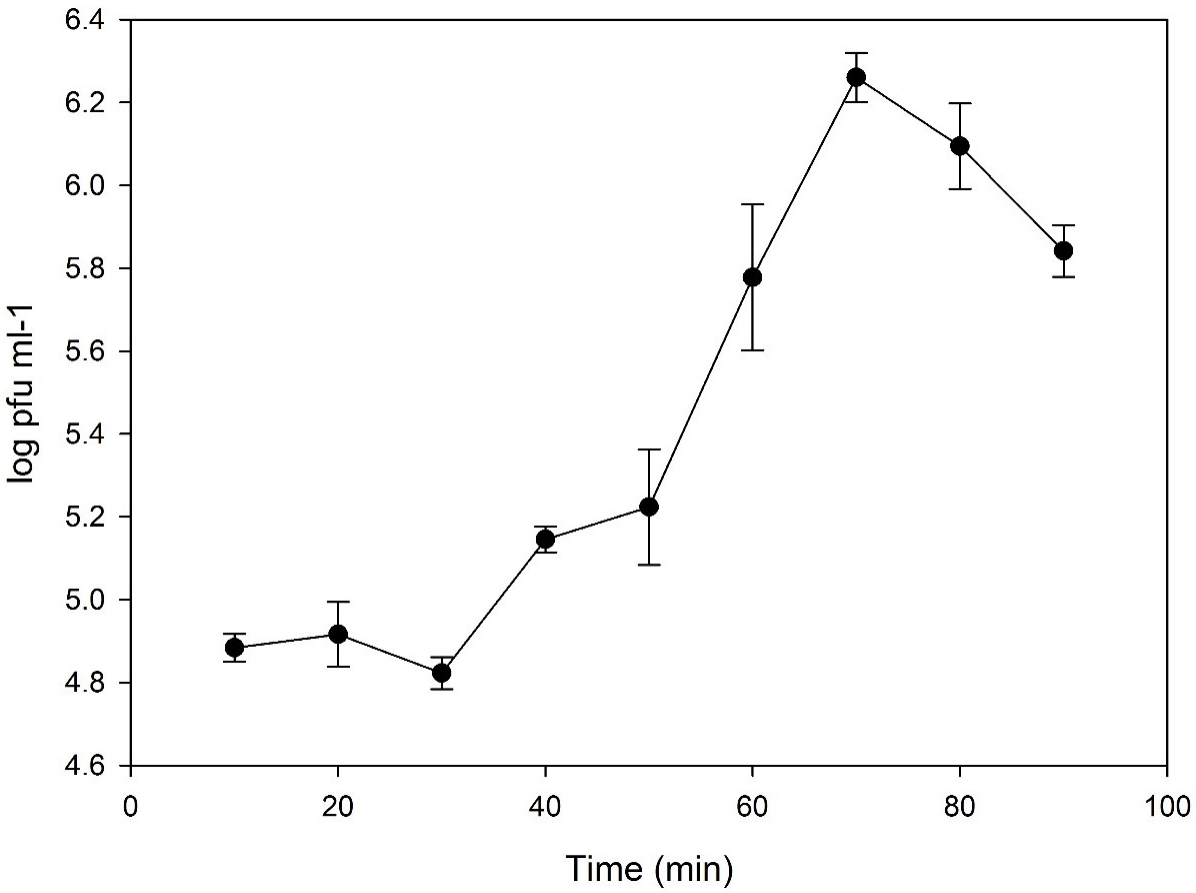
One-step growth curve of bacteriophage pVa5. The values are means ± standard deviation of three replicates.

One-step growth curve analysis (Fig 2) showed that pVa5 had a latent period of 30 min and a burst size of 24 virions per infected cell. N4-like bacteriophages demonstrate a great variability in both these life cycle parameters. Latency periods between 10–15 min to 6h and burst sizes between 10 to even 9000 virions per cell have already been reported in the literature. For instance, in the case of phiAxp-3 a latency time of 80 min and burst size of 9000 virions per infected cell have been observed [35–37].

A spot test showed that the phage has a very limited lytic spectrum based on the 15 tested strains, since it could infect only the *V*. *splendidus* host strain VaAn and no other *Vibrio splendidus* strains, nor *Vibrio* species tested for phage susceptibility (Table 1).

**Fig 3:**
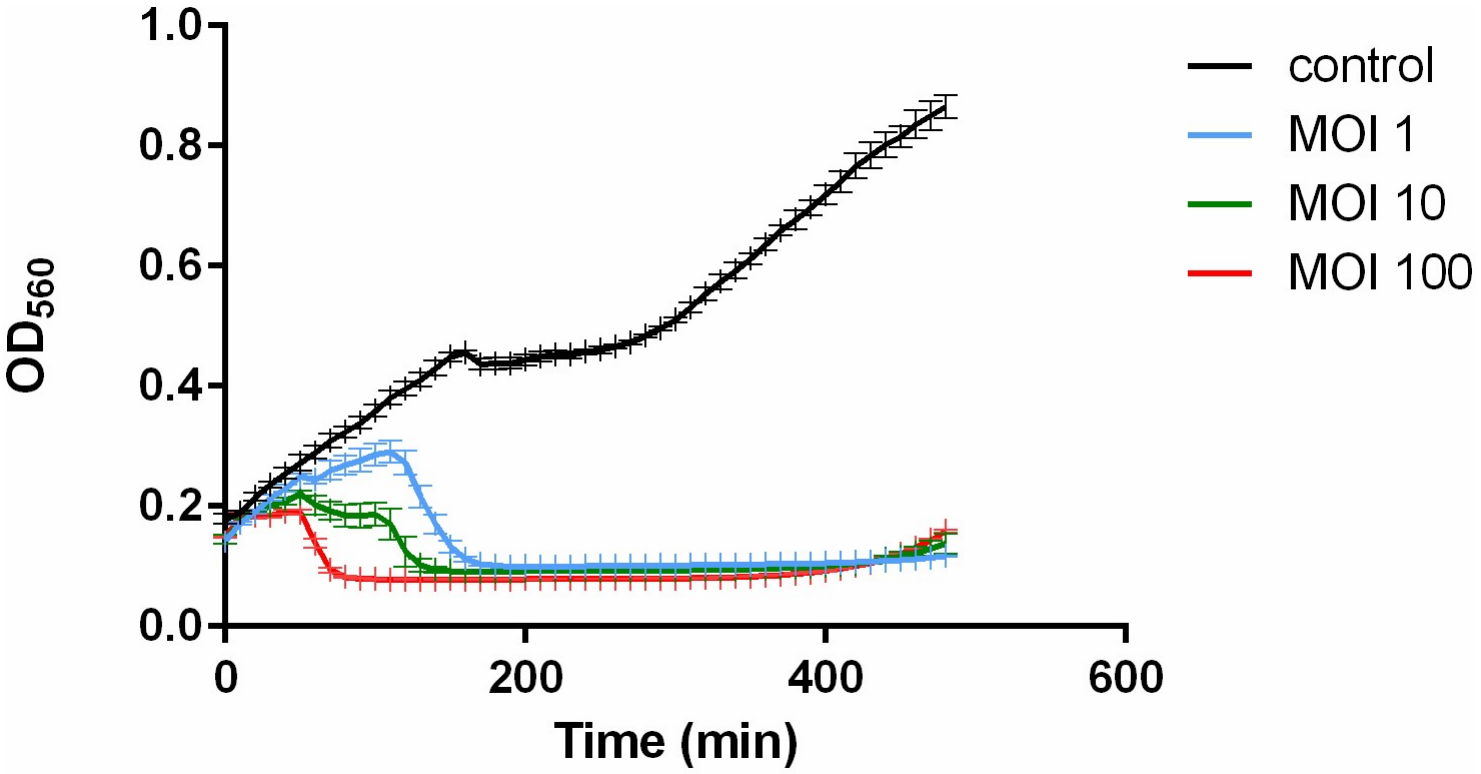
*In vitro* lysis assay using bacteriophage pVa5 and the host bacterium *V*. *splendidus* VaAn at 3 different MOIs at 25°C. The values are means ± standard deviation of 3 replicates.

*In vitro* lysis assay indicated that pVa5 could almost completely eliminate bacterial growth for 8 hours (Fig 3). The intense bactericidal activity that the phage demonstrated, renders it a very powerful candidate for *in vivo* phage therapy trials against *V*. *splendidus* strain VaAn. The inhibition of bacterial growth was equally marked with all three different applied MOIs, even at MOI: 1. The high efficiency of the phage makes it also practically convenient for use even in large volumes of water, such as fish and shellfish hatcheries, which would eventually be the ultimate goal. The regrowth of the bacterial population which takes place after almost 8h, even though it is very slow, could be considered as a constraint to phage therapy application. However, it has been adequately reported in the literature that bacteriophage resistance is associated with reduced virulence [38,39]. Hence, the exact effect of resistance development needs to be further evaluated through meticulously designed *in vivo* trials.

### Genomic characterization

The genome of pVa5 had a size of 78,145 bp and a G+C content of 43.2% which is comparable to the host G+C content (44.3%). 107 genes were identified (S1 Table) of which 101 had ATG as starting codon, 4 GTG genes: 40.2, 45, 47 and 55) and 2 TTG (genes: 33 and 36). The coding region size of the genome was 71,258 bp (91.18% of the whole genome).

**Fig 4:**
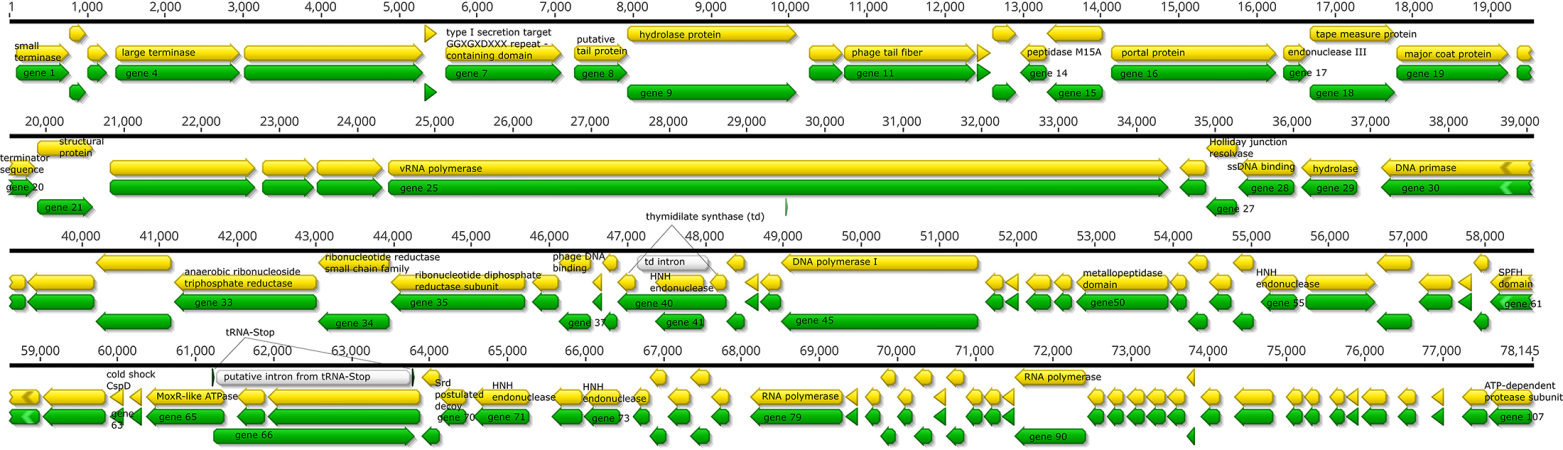
Gene map of pVa5. Genes with attributed function are noted in the figure. Yellow: CDS, Green: genes, White: introns, Dark green: putative tRNA.

The digestion of the viral genome with ApaI produced four distinct DNA fragments, which clearly indicated that the genome contains three single restriction sites. ApaI recognizes and cleaves GGGCC^C genomic sites and the presence of three such sites were *in silico* confirmed in the phage genome. If the genome were circular, we should expect to find three distinct DNA fragments; however in our case, ApaI treatment yielded four distinct DNA fragments. Hence, it can be concluded that the physical structure of the pVa5 phage genome is linear and not circular. However, specific PCR using two sets of primers that match the start (bp 1) and end (bp 78,145) point of the physical phage genome, was able in both sets to amplify the targeted genomic parts. In physically-linear genome phages, the packaging mechanism during replication may explain this result. Circularly permuted genomes that follow the formation of a linear concatemer during replication packaging do not have defined ends. Similarly, in our case, the replication process of pVa5 ensures the successful packaging of a complete 107-gene set (Fig 4) in each of the newly produced virions [40]; however, the DNA molecule is not always packed with the same start and end positions. A hydrolase CDS (gene 9) was also detected as soon as the obtained phage genome contig was *in silico* circularized, corroborating the circularly permuted propagation strategy of the pVa5. According to the convention followed for the circularly permuted phages, bp1 is set as the first nucleotide of the closest intergenic region immediately to the left of the terminase gene. In our case, the small terminase gene (gene 1) and large terminase gene (gene 4) were annotated as gp69 and gp68 since they resembled, respectively, the originally annotated small and large terminase genes of the N4 phage [41] by 61.7% and 71.2%.

None of the tRNA detection bioinformatic tools used were able to identify any tRNA genes, indicating that the phage is adapted to the host tRNA makeup for its protein synthesis [42]. However, after modifying the default parameters of the ARAGORN software (Allow introns 0–3000 bases: yes), an intervening ~2.5 kbp coding sequence (CDS) containing genomic area was present in a tRNA^Stop^ gene. Bacteriophages often carry tRNA genes for the regulation of translation-associated functions [43,44]. Even though intron-mediated tRNAs have been reported in both eukaryotic nuclei and Archaea [45], this finding is, to our knowledge, the first observed in a bacteriophage genome. This tRNA^Stop^ gene matches to the TAA stop codon and since TAA is the prevalent stop codon in pVa5 genome (72 out of 107 CDS), it could potentially contribute to regulatory procedures during viral infection. It has to be stated however that although the genomic structure of the tRNA gene matches to a tRNAStop, this identification might be an artifact. In order to be certain about its existence and its functionality, the specific mechanism of this gene’s function still needs to be elucidated.

Analysis of the pVa5 nucleotide sequence using the BLASTn algorithm of the NCBI Database [46] showed that the novel phage is unique as its closest similarity is with *Pseudoalteromonas* phage pYD6-A (E-value, 0.0; query coverage, 5%; identity, 78%) and the vibriophages VPB32 and VPB47 (E-value, 0.0; query coverage, 7%; identity, 76%) all of which are also N4-like podoviruses. Individual-gene BLAST search led to 34 genes with e-value > 0.01 and query coverage > 30% (S1 Table). The novelty of pVa5 is also supported by the fact that 29% of its total genes (31 out of 34), correspond to their homologues in pYD6-A, VPB32 and VPB47. After mapping each of the pVa5 phage genes against the nr nucleotide collection of NCBI, no relationship with any potential virulence or fitness factor was documented. The structure of the proteins or hypothetical proteins did not either indicate any correlations with known toxins, corroborating to the lytic nature of the bacteriophage. After whole genome comparison on the amino acid level, pVa5 shared 24%, 22.7% and 14.4% identical sites with VBP47, VBP32 and pYD6-A, respectively. However, when the comparison was focused on the homologues, the pairwise alignement percentages ranged from at least 50% up to 90%. Our data suggest that pVa5 is no lysogenic like all other N4-like phages which have been recorded so far.

The key features characterizing the N4-like phages are the presence of a very large virion-encapsulated DNA-dependent RNA polymerase (vRNAP) responsible for the early gene transcription, 2 RNA polymerases and a single stranded DNA-binding protein [47,48]. The vRNAP of pVa5 (gene 25) is 3,345 aa in size, while the phage contains two more RNA polymerases (gene 79 and gene 90) 405 and 311 aa in size. The phage-encoded RNAPs of pVa5 are able, as in all N4-like phages, to establish the transcriptional independence of the virus [49]. Gene 28 was predicted to encode the single-stranded DNA binding (SSB) protein for pVa5. In the N4 phage, the SSB acts as an architectural transcription factor by providing an active promoter conformation for vRNAP binding [50].

The genomic organization of N4-like phages is considered to be highly conserved [42,51]. The genomes of N4-like phages are organized basically in two large clusters transcribed in opposite directions [52]; this type of organization is followed by pVa5, nevertheless with two exceptions, genes 14 and 15, which encode a peptidase M15A related to lysis and a phosphate starvation-inducible protein (PhoH) (possibly a phosphorus regulation gene). It has been demonstrated that many marine phages contain genes related to pho regulation [53] and although its function remains unclear, there has been speculation that the phages might use these genes to take over the phosphorus acquisition mechanism for their own benefit during phage DNA replication [54]. Similarly, ribonucleotide reductase (RNR), the enzyme responsible for the formation of deoxyribonucleotides from ribonucleotides, is also considered to be a tool used by the phage to sustain host metabolism promoting its own replication [55]. Bacteriophage pVa5 has three consecutive genes organized in a cluster (genes 33–35) encoding RNRs belonging to class I and class III which is not very common in podoviridae [56]. One of the most interesting findings in the genome of pVa5 is the presence of an intervening sequence containing an HNH endonuclease (gene 41) splicing the thymidylate synthase (td) gene (CDS 40.1 and 40.2). The concatenated amino acid sequence of the two thymidylate synthase CDS of pVa5 was aligned and mapped against their homologue td synthase genes from the bacteriophages VBP32, VBP47 and pYD6-A (S2 Fig). Apart from a 57 aa sequence that was completely missing from the td synthase gene of pVa5, their pairwise percentage identity was estimated at 82.8%, indicating a very high degree of conservation. Thymidylate synthase (*td*) is one of the most conserved enzymes among bacteriophage genomes [57] and partition of the gene by an intron into two separate genes has been observed on two other occasions: in the *E*. *coli* bacteriophage T4 [58] and in *Bacillus* bacteriophage b22 [59]. It has been suggested that *td* has a dual role related to catalysis and to the structure of phage baseplate, thus a differential expression that might be facilitated by the splicing could be the underlying reason [58]. Additionally, the td synthase intron is characterized as a group I intron (E-value: 1E-18, Accession: RF00028) in the Rfam database [33]. Group I introns are self-splicing introns that even though initially considered as molecular parasites, have been found to act in a co-evolution context which promotes mutualistic interactions with their host genes [60]. The first reported microbial group I intron was the intron that was also found in the td gene of the T4 bacteriophage [58]. Several group I introns found in bacteriophage genomes are being reported in the literature with an increasing frequency [61,62]. Their self-splicing nature allows them to act as ribozymes catalyzing the expression of the gene in which they have intervened. Furthermore, the homing (HNH) endonuclease genes that are usually encoded by such introns, allow the introns to act also as mobile genetic elements and expand their distribution in intron-less genes through DNA-based recombination mobility [63,64]. This finding is also in accordance to the rules that should be followed by group I intron ribozymes [65]: 1) the pVa5 group I intron is located in a highly conserved gene (td gene); 2) it has a T preceding the insertion site and a G as last nucleotide of the insertion; 3) the size of the intron without the HNH endonuclease encoding area is 279 nt. However, it should be stated that HNH endonucleases are quite common, not only in the N4-like phages but generally in phage genomes since they constitute widespread components of phage DNA packaging machinery [66].

**Fig 5:**
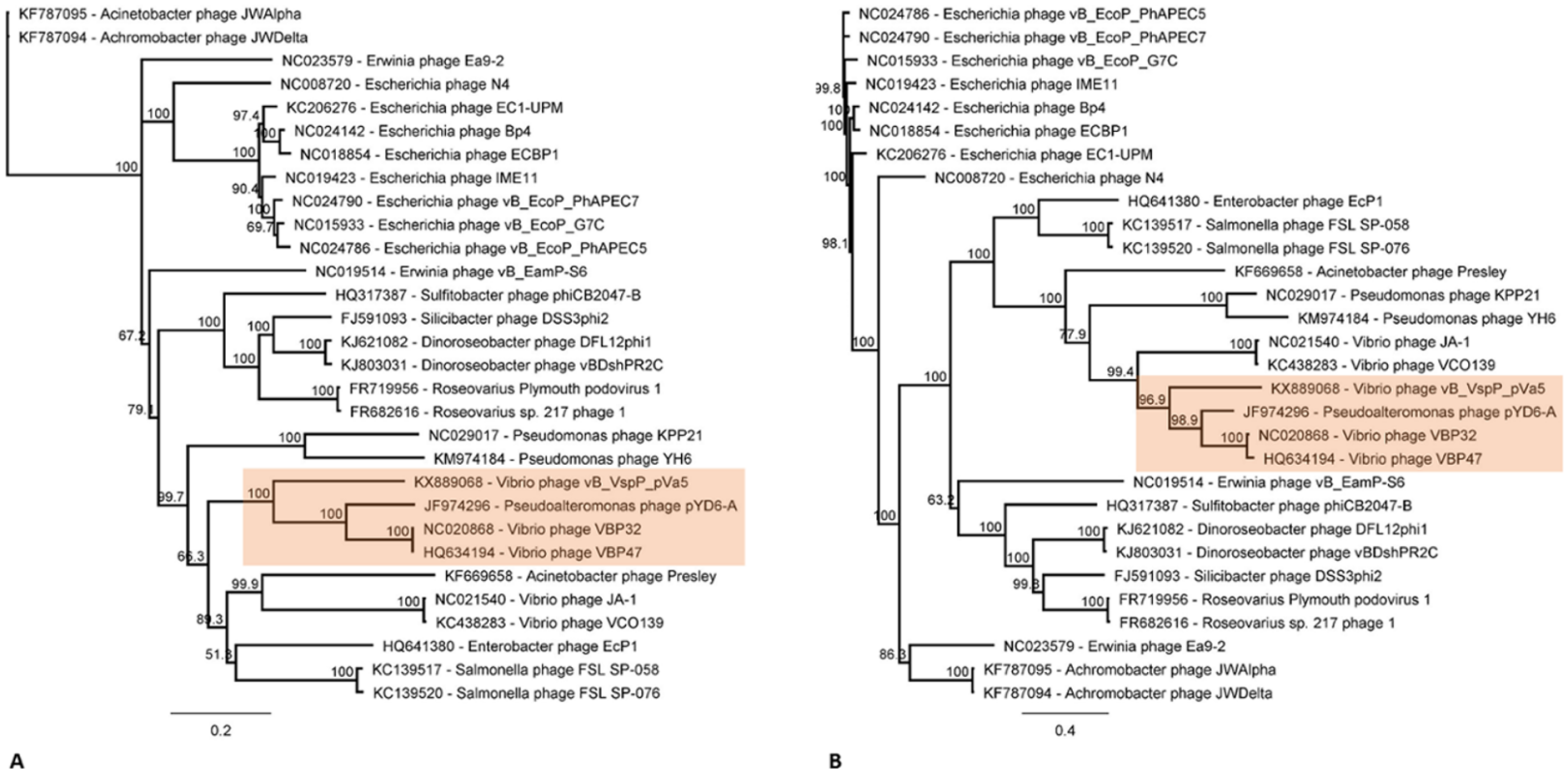
Molecular phylogenetic analysis by Neighbor-joining method based on the Tamura-Nei model. The consensus tree which was produced by 1000 bootstraps is shown. The novel bacteriophage vB_VspP_pVa5 is classified by two different genetic markers: DNA Polymerase I (**A**) and virion-encapsulated RNA Polymerase (**B**) in a statistically robust taxonomic group which is highlighted by green areas and includes two *Vibrio* phages (NC020868 and HQ634194) and one *Pseudoalteromonas* phage (JF974296). The analysis involved 30 nucleotide sequences.

**Fig 6:**
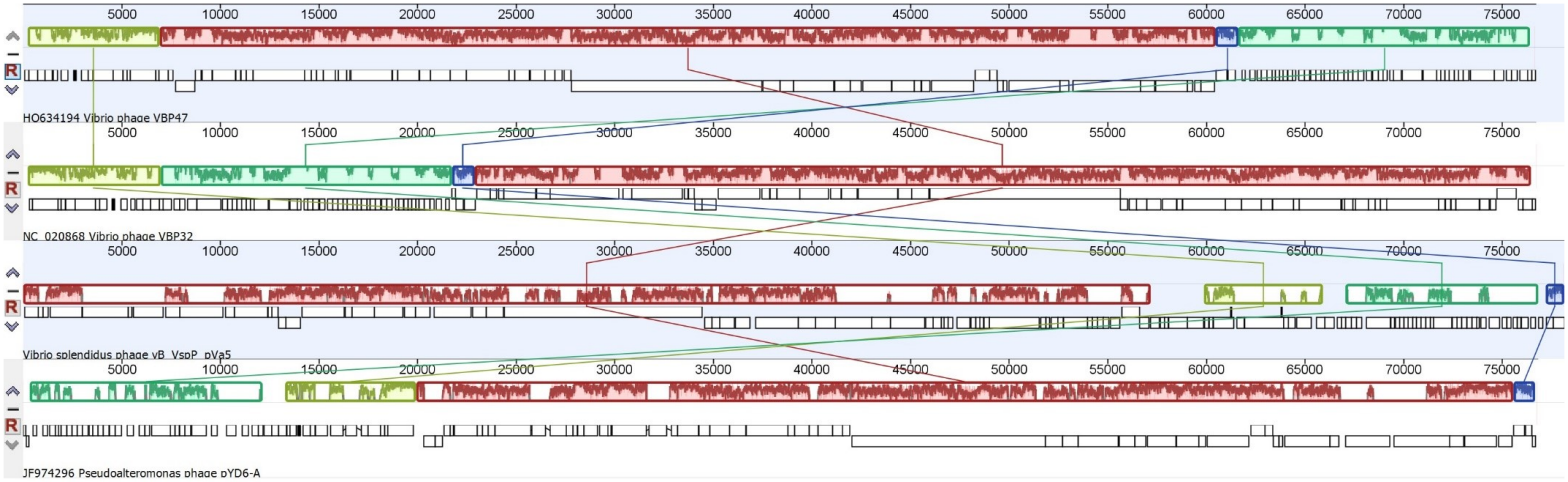
Multiple alignment of the whole genomes of all N4-like Vibriophages using the progressive Mauve algorithm. Same coloured blocks indicate high synteny between genomes without genomic rearrangements. Genomic similarity is represented by the height of the bars, which correspond to the average level of conservation in that region of the genome sequence. Completely white regions represent fragments that were not aligned or contained sequence elements specific to a particular genome. The four bacteriophages included in the analysis are classified in the same taxonomic group by both DNAP and vRNAP gene markers.

The lysis cassette of N4-like phages typically contains an endolysin, an N-acetylmuramoyl-L-alanine amidase, a holin and a peptidoglycan-degrading protein [52]. In pVa5 no lysis cluster could be identified and the only protein that could be related to lysis was a peptidase M15 encoded by gene 14. Similarly, a lysis cluster was also absent in the phages of *Vibrio parahaemolyticus* VBP32, VBP47 and *Pseudoalteromonas phage* pYD6-A [52]. Interestingly, these phages were evolutionarily closer to pVa5 as demonstrated by the phylogeny based on DNAP and vRNAP genes (Fig 5). Multiple alignment of the whole genomes of these phages using the progressive Mauve algorithm showed that pVa5 shares similar synteny characteristics with its taxonomic group phages (Fig 6). In addition to the closely related N4-like vibrio phages VBP47 and VBP32 which are identical and infect *Vibrio parahaemolyticus* strain RIMD2210633, the two other sequenced N4-like phages Ja-1 and VCO139 which infect *Vibrio cholerae* [67] were phylogenetically clustered in different but still vicinal branches. Especially in the RNAP-based phylogeny, all Vibriophages are clustered into the same taxonomic group which is then further divided into two distinct but closely related branches (Fig 5B).

## Conclusions

Bacteriophage vB_VspP_pVa5 is a novel virulent podovirus that infects *Vibrio splendidus*. Although it is a virulent phage based on the genomic analysis, it has a narrow host range and therefore is not suitable for a universal phage therapy scheme. On the other hand, it has interesting genomic features that significantly contribute to our current knowledge about N4-like phages and could therefore be exploited in phage infection studies in combination with the genomic information available for its bacterial host.

## Supporting information

S1 Table Annotation of the vB_VspP_pVa5 Vibriophage accompanied by the BLAST results of the genes. Genes with e-value > 0.01 and query coverage > 30% were omitted from the BLAST results in order to prevent a false interpretation of the results.
(DOCX)

S1 Fig Four sets of primers manually designed; sets 1 and 2 were able to amplify the genomic area between the first and last bp, whereas sets pos1 and pos2 served as positive controls in order to confirm the functionality of the PCR. The relative position of the primers as well as the genomic parts expected to be amplified, are also schematically depicted below.
(PDF)

S2 Fig Pairwise alignment and comparison of the amino acid sequences of the 4 thymidylate synthase genes from 4 N4-like phages classified in the same taxonomic group: *Vibrio* phage VBP47 (HQ634194), *Vibrio* phage VBP32 (NC_020868), *Pseudoalteromonas* phage pYD6-a (JF974296) and *Vibrio* phage vB_VspP_pVa5 (KX889068). (PDF)
