# Supplementary material for: Isolation and characterization of a N4-like lytic bacteriophage infecting Vibrio splendidus, a pathogen of fish and bivalves
Source: PLoS One. 2017 Dec 28;12(12):e0190083. doi: 10.1371/journal.pone.0190083 (PMC5746245; doi:10.1371/journal.pone.0190083)
Supplement: S1 Table — Genes with e-value > 0.01 and query coverage > 30% were omitted from the BLAST results in order to prevent a false interpretation of the results. (DOCX) [file pone.0190083.s001.docx]

| Genes with CDS | Type | Start bp | End bp | Length | Direction |
| --- | --- | --- | --- | --- | --- |
| N4 gp69 like CDS | CDS | 86 | 781 | 696 | forward |
| Gene 1 | gene | 86 | 781 | 696 | forward |
| hypothetical protein CDS | CDS | 768 | 1,013 | 246 | forward |
| Gene 2 | gene | 768 | 1,013 | 246 | forward |
| hypothetical protein CDS | CDS | 1,006 | 1,275 | 270 | forward |
| Gene 3 | gene | 1,006 | 1,275 | 270 | forward |
| N4 gp68 like CDS | CDS | 1,368 | 2,972 | 1,605 | forward |
| Gene 4 | gene | 1,368 | 2,972 | 1,605 | forward |
| hypothetical protein CDS | CDS | 3,014 | 5,329 | 2,316 | forward |
| Gene 5 | gene | 3,014 | 5,329 | 2,316 | forward |
| hypothetical protein CDS | CDS | 5,326 | 5,505 | 180 | forward |
| Gene 6 | gene | 5,326 | 5,505 | 180 | forward |
| type I secretion target GGXGXDXXX repeat-containing domain , partial CDS | CDS | 5,604 | 7,097 | 1,494 | forward |
| Gene 7 | gene | 5,604 | 7,097 | 1,494 | forward |
| Putative tail protein CDS | CDS | 7,240 | 7,947 | 708 | forward |
| Gene 8 | gene | 7,240 | 7,947 | 708 | forward |
| Hydrolase protein CDS | CDS | 7,938 | 10,118 | 2,181 | forward |
| Gene 9 | gene | 7,938 | 10,118 | 2,181 | forward |
|  | terminator | 10,180 | 10,218 | 39 | forward |
| hypothetical protein CDS | CDS | 10,259 | 10,708 | 450 | forward |
| Gene 10 | gene | 10,259 | 10,708 | 450 | forward |
| Phage tail fiber CDS | CDS | 10,719 | 12,407 | 1,689 | forward |
| Gene 11 | gene | 10,719 | 12,407 | 1,689 | forward |
| hypothetical protein CDS | CDS | 12,422 | 12,610 | 189 | forward |
| Gene 12 | gene | 12,422 | 12,610 | 189 | forward |
| hypothetical protein CDS | CDS | 12,610 | 12,927 | 318 | forward |
| Gene 13 | gene | 12,610 | 12,927 | 318 | forward |
|  | terminator | 12,921 | 12,968 | 48 | forward |
| Peptidase M15A | CDS | 12,963 | 13,325 | 363 | reverse |
| Gene 14 | gene | 12,963 | 13,325 | 363 | reverse |
| PhoH-like protein CDS | CDS | 13,309 | 14,052 | 744 | reverse |
| Gene 15 | gene | 13,309 | 14,052 | 744 | reverse |
| Portal protein, N4 gp59 CDS | CDS | 14,141 | 16,276 | 2,136 | forward |
| Gene16 | gene | 14,141 | 16,276 | 2,136 | forward |
| Endonuclease III CDS | CDS | 16,340 | 16,678 | 339 | forward |
| Gene 17 | gene | 16,340 | 16,678 | 339 | forward |
| Tape measure protein | CDS | 16,678 | 17,793 | 1,116 | forward |
| Gene 18 | gene | 16,678 | 17,793 | 1,116 | forward |
| Major coat protein CDS | CDS | 17,805 | 19,253 | 1,449 | forward |
| Gene 19 | gene | 17,805 | 19,253 | 1,449 | forward |
| Terminator sequence | terminator | 19,261 | 19,301 | 41 | forward |
| putative N4 g55 CDS | CDS | 19,340 | 19,891 | 552 | forward |
| Gene 20 | gene | 19,340 | 19,891 | 552 | forward |
| Structural protein CDS | CDS | 19,891 | 20,625 | 735 | forward |
| Gene 21 | gene | 19,891 | 20,625 | 735 | forward |
| N4 gp53 CDS | CDS | 20,836 | 22,710 | 1,875 | forward |
| Gene 22 | gene | 20,836 | 22,710 | 1,875 | forward |
| hypothetical protein CDS | CDS | 22,782 | 23,471 | 690 | forward |
| Gene 23 | gene | 22,782 | 23,471 | 690 | forward |
| hypothetical protein CDS | CDS | 23,482 | 24,339 | 858 | forward |
| Gene 24 | gene | 23,482 | 24,339 | 858 | forward |
| vRNA polymerase CDS | CDS | 24,392 | 34,426 | 10,035 | forward |
| Gene 25 | gene | 24,392 | 34,426 | 10,035 | forward |
| hypothetical protein CDS | CDS | 34,551 | 34,916 | 366 | reverse |
| Gene 26 | gene | 34,551 | 34,916 | 366 | reverse |
| Holliday junction resolvase CDS | CDS | 34,891 | 35,310 | 420 | reverse |
| Gene 27 | gene | 34,891 | 35,310 | 420 | reverse |
| Single-stranded DNA-binding CDS | CDS | 35,310 | 36,050 | 741 | reverse |
| Gene 28 | gene | 35,310 | 36,050 | 741 | reverse |
| Hydrolase protein, N4 gp44 CDS | CDS | 36,117 | 36,842 | 726 | reverse |
| Gene 29 | gene | 36,117 | 36,842 | 726 | reverse |
| DNA primase CDS | CDS | 37,135 | 39,300 | 2,166 | reverse |
| Gene 30 | gene | 37,135 | 39,300 | 2,166 | reverse |
| hypothetical protein CDS | CDS | 39,312 | 40,187 | 876 | reverse |
| Gene 31 | gene | 39,312 | 40,187 | 876 | reverse |
| N4 gp42 CDS | CDS | 40,187 | 41,170 | 984 | reverse |
| Gene 32 | gene | 40,187 | 41,170 | 984 | reverse |
| Anaerobic ribonucleoside triphosphate reductase CDS | CDS | 41,183 | 43,039 | 1,857 | reverse |
| Gene 33 | gene | 41,183 | 43,039 | 1,857 | reverse |
| Ribonucleotide reductase, small chain family CDS | CDS | 43,036 | 43,977 | 942 | reverse |
| Gene 34 | gene | 43,036 | 43,977 | 942 | reverse |
| Ribonucleotide-diphosphate reductase subunit alpha CDS | CDS | 43,965 | 45,719 | 1,755 | reverse |
| Gene 35 | gene | 43,965 | 45,719 | 1,755 | reverse |
| hypothetical protein CDS | CDS | 45,777 | 46,136 | 360 | reverse |
| Gene 36 | gene | 45,777 | 46,136 | 360 | reverse |
| Phage DNA binding CDS | CDS | 46,133 | 46,555 | 423 | reverse |
| Gene 37 | gene | 46,133 | 46,555 | 423 | reverse |
| hypothetical protein CDS | CDS | 46,548 | 46,691 | 144 | reverse |
| Gene 38 | gene | 46,548 | 46,691 | 144 | reverse |
| hypothetical protein CDS | CDS | 46,681 | 46,890 | 210 | reverse |
| Gene 39 | gene | 46,681 | 46,890 | 210 | reverse |
| Thymidylate synthase CDS 2 | CDS | 46,878 | 47,132 | 255 | reverse |
| Gene 40 | gene | 46,878 | 48,287 | 1,410 | reverse |
| Thymidilate synthase intron | intron | 47,133 | 48,068 | 936 | none |
| HNH endonuclease CDS | CDS | 47,361 | 48,017 | 657 | reverse |
| Gene 41 | gene | 47,361 | 48,017 | 657 | reverse |
| Thymidylate synthase CDS 1 | CDS | 48,069 | 48,287 | 219 | reverse |
| hypothetical protein CDS | CDS | 48,287 | 48,529 | 243 | reverse |
| Gene 42 | gene | 48,287 | 48,529 | 243 | reverse |
| hypothetical protein CDS | CDS | 48,510 | 48,704 | 195 | reverse |
| Gene 43 | gene | 48,510 | 48,704 | 195 | reverse |
| hypothetical protein CDS | CDS | 48,714 | 48,995 | 282 | reverse |
| Gene 44 | gene | 48,714 | 48,995 | 282 | reverse |
| DNA polymerase I | CDS | 48,979 | 51,522 | 2,544 | reverse |
| Gene 45 | gene | 48,979 | 51,522 | 2,544 | reverse |
| hypothetical protein CDS | CDS | 51,591 | 51,854 | 264 | reverse |
| Gene 46 | gene | 51,591 | 51,854 | 264 | reverse |
| hypothetical protein CDS | CDS | 51,856 | 52,041 | 186 | reverse |
| Gene 47 | gene | 51,856 | 52,041 | 186 | reverse |
| hypothetical protein CDS | CDS | 52,116 | 52,454 | 339 | reverse |
| Gene 48 | gene | 52,116 | 52,454 | 339 | reverse |
| hypothetical protein CDS | CDS | 52,470 | 52,730 | 261 | reverse |
| Gene 49 | gene | 52,470 | 52,730 | 261 | reverse |
| Metallopeptidase domain CDS | CDS | 52,764 | 53,960 | 1,197 | reverse |
| Gene 50 | gene | 52,764 | 53,960 | 1,197 | reverse |
| hypothetical protein CDS | CDS | 53,963 | 54,196 | 234 | reverse |
| Gene 51 | gene | 53,963 | 54,196 | 234 | reverse |
| hypothetical protein CDS | CDS | 54,196 | 54,468 | 273 | reverse |
| Gene 52 | gene | 54,196 | 54,468 | 273 | reverse |
| hypothetical protein CDS | CDS | 54,465 | 54,776 | 312 | reverse |
| Gene 53 | gene | 54,465 | 54,776 | 312 | reverse |
| hypothetical protein CDS | CDS | 54,773 | 55,057 | 285 | reverse |
| Gene 54 | gene | 54,773 | 55,057 | 285 | reverse |
| HNH homing endonuclease protein | CDS | 55,133 | 55,615 | 483 | reverse |
| Gene 55 | gene | 55,133 | 55,615 | 483 | reverse |
| hypothetical protein CDS | CDS | 55,700 | 56,620 | 921 | forward |
| Gene 56 | gene | 55,700 | 56,620 | 921 | forward |
| hypothetical protein CDS | CDS | 56,617 | 57,093 | 477 | reverse |
| Gene 57 | gene | 56,617 | 57,093 | 477 | reverse |
| hypothetical protein CDS | CDS | 57,161 | 57,598 | 438 | reverse |
| Gene 58 | gene | 57,161 | 57,598 | 438 | reverse |
| hypothetical protein CDS | CDS | 57,654 | 57,866 | 213 | reverse |
| Gene 59 | gene | 57,654 | 57,866 | 213 | reverse |
| hypothetical protein CDS | CDS | 57,866 | 58,069 | 204 | reverse |
| Gene 60 | gene | 57,866 | 58,069 | 204 | reverse |
| SPFH domain CDS | CDS | 58,082 | 59,023 | 942 | reverse |
| Gene 61 | gene | 58,082 | 59,023 | 942 | reverse |
| hypothetical protein CDS | CDS | 59,048 | 59,863 | 816 | reverse |
| Gene 62 | gene | 59,048 | 59,863 | 816 | reverse |
| cold shock CspD CDS | CDS | 59,898 | 60,104 | 207 | reverse |
| Gene 63 | gene | 59,898 | 60,104 | 207 | reverse |
| hypothetical protein CDS | CDS | 60,150 | 60,335 | 186 | reverse |
| Gene 64 | gene | 60,150 | 60,335 | 186 | reverse |
| MoxR-like ATPase CDS | CDS | 60,367 | 61,386 | 1,020 | reverse |
| Gene 65 | gene | 60,367 | 61,386 | 1,020 | reverse |
| potential tRNA-Stop(tta) | tRNA | 61,223 | 61,258 | 36 | forward |
| Gene 66 - putative tRNA-Stop(tta) | gene | 61,223 | 63,832 | 2,610 | forward |
| putative Intron from tRNA-stop(tta) | intron | 61,259 | 63,772 | 2,514 | none |
| hypothetical protein CDS | CDS | 61,543 | 61,920 | 378 | reverse |
| Gene 67 | gene | 61,543 | 61,920 | 378 | reverse |
| hypothetical protein CDS | CDS | 61,930 | 63,909 | 1,980 | reverse |
| Gene 68 | gene | 61,930 | 63,909 | 1,980 | reverse |
| potential tRNA-Stop(tta) | tRNA | 63,773 | 63,832 | 60 | forward |
| hypothetical protein CDS | CDS | 63,909 | 64,157 | 249 | reverse |
| Gene 69 | gene | 63,909 | 64,157 | 249 | reverse |
| Srd postulated decoy CDS | CDS | 64,132 | 64,500 | 369 | reverse |
| Gene 70 | gene | 64,132 | 64,500 | 369 | reverse |
| HNH homing endonuclease CDS | CDS | 64,566 | 65,303 | 738 | reverse |
| Gene 71 | gene | 64,566 | 65,303 | 738 | reverse |
| hypothetical protein CDS | CDS | 65,570 | 65,986 | 417 | reverse |
| Gene 72 | gene | 65,570 | 65,986 | 417 | reverse |
| HNH homing endonuclease CDS | CDS | 65,990 | 66,472 | 483 | reverse |
| Gene 73 | gene | 65,990 | 66,472 | 483 | reverse |
| hypothetical protein CDS | CDS | 66,622 | 66,840 | 219 | reverse |
| Gene 74 | gene | 66,622 | 66,840 | 219 | reverse |
| hypothetical protein CDS | CDS | 66,833 | 67,060 | 228 | reverse |
| Gene 75 | gene | 66,833 | 67,060 | 228 | reverse |
| hypothetical protein CDS | CDS | 67,053 | 67,361 | 309 | reverse |
| Gene 76 | gene | 67,053 | 67,361 | 309 | reverse |
| hypothetical protein CDS | CDS | 67,354 | 67,617 | 264 | reverse |
| Gene 77 | gene | 67,354 | 67,617 | 264 | reverse |
| hypothetical protein CDS | CDS | 67,617 | 67,874 | 258 | reverse |
| Gene 78 | gene | 67,617 | 67,874 | 258 | reverse |
| RNA polymerase CDS | CDS | 68,114 | 69,328 | 1,215 | reverse |
| Gene 79 | gene | 68,114 | 69,328 | 1,215 | reverse |
| hypothetical protein CDS | CDS | 69,340 | 69,522 | 183 | reverse |
| Gene 80 | gene | 69,340 | 69,522 | 183 | reverse |
| hypothetical protein CDS | CDS | 69,584 | 69,802 | 219 | reverse |
| Gene 81 | gene | 69,584 | 69,802 | 219 | reverse |
| hypothetical protein CDS | CDS | 69,789 | 70,007 | 219 | reverse |
| Gene 82 | gene | 69,789 | 70,007 | 219 | reverse |
| hypothetical protein CDS | CDS | 70,000 | 70,224 | 225 | reverse |
| Gene 83 | gene | 70,000 | 70,224 | 225 | reverse |
| hypothetical protein CDS | CDS | 70,221 | 70,472 | 252 | reverse |
| Gene 84 | gene | 70,221 | 70,472 | 252 | reverse |
| hypothetical protein CDS | CDS | 70,465 | 70,647 | 183 | reverse |
| Gene 85 | gene | 70,465 | 70,647 | 183 | reverse |
| hypothetical protein CDS | CDS | 70,640 | 70,882 | 243 | reverse |
| Gene 86 | gene | 70,640 | 70,882 | 243 | reverse |
| hypothetical protein CDS | CDS | 70,875 | 71,108 | 234 | reverse |
| Gene 87 | gene | 70,875 | 71,108 | 234 | reverse |
| hypothetical protein CDS | CDS | 71,110 | 71,349 | 240 | reverse |
| Gene 88 | gene | 71,110 | 71,349 | 240 | reverse |
| hypothetical protein CDS | CDS | 71,351 | 71,524 | 174 | reverse |
| Gene 89 | gene | 71,351 | 71,524 | 174 | reverse |
| RNA polymerase CDS | CDS | 71,514 | 72,446 | 933 | reverse |
| Gene 90 | gene | 71,514 | 72,446 | 933 | reverse |
| hypothetical protein CDS | CDS | 72,449 | 72,685 | 237 | reverse |
| Gene 91 | gene | 72,449 | 72,685 | 237 | reverse |
| hypothetical protein CDS | CDS | 72,687 | 72,926 | 240 | reverse |
| Gene 92 | gene | 72,687 | 72,926 | 240 | reverse |
| hypothetical protein CDS | CDS | 72,938 | 73,189 | 252 | reverse |
| Gene 93 | gene | 72,938 | 73,189 | 252 | reverse |
| hypothetical protein CDS | CDS | 73,191 | 73,460 | 270 | reverse |
| Gene 94 | gene | 73,191 | 73,460 | 270 | reverse |
| hypothetical protein CDS | CDS | 73,462 | 73,719 | 258 | reverse |
| Gene 95 | gene | 73,462 | 73,719 | 258 | reverse |
| hypothetical protein CDS | CDS | 73,712 | 73,840 | 129 | reverse |
| Gene 96 | gene | 73,712 | 73,840 | 129 | reverse |
| hypothetical protein CDS | CDS | 73,900 | 74,172 | 273 | reverse |
| Gene 97 | gene | 73,900 | 74,172 | 273 | reverse |
| hypothetical protein CDS | CDS | 74,334 | 74,849 | 516 | reverse |
| Gene 98 | gene | 74,334 | 74,849 | 516 | reverse |
| hypothetical protein CDS | CDS | 74,999 | 75,229 | 231 | reverse |
| Gene 99 | gene | 74,999 | 75,229 | 231 | reverse |
| hypothetical protein CDS | CDS | 75,231 | 75,437 | 207 | reverse |
| Gene 100 | gene | 75,231 | 75,437 | 207 | reverse |
| hypothetical protein CDS | CDS | 75,543 | 75,767 | 225 | reverse |
| Gene 101 | gene | 75,543 | 75,767 | 225 | reverse |
| hypothetical protein CDS | CDS | 75,769 | 75,948 | 180 | reverse |
| Gene 102 | gene | 75,769 | 75,948 | 180 | reverse |
| hypothetical protein CDS | CDS | 75,960 | 76,307 | 348 | reverse |
| Gene 103 | gene | 75,960 | 76,307 | 348 | reverse |
| hypothetical protein CDS | CDS | 76,435 | 76,677 | 243 | reverse |
| Gene 104 | gene | 76,435 | 76,677 | 243 | reverse |
| hypothetical protein CDS | CDS | 76,853 | 77,041 | 189 | reverse |
| Gene 105 | gene | 76,853 | 77,041 | 189 | reverse |
| hypothetical protein CDS | CDS | 77,246 | 77,584 | 339 | reverse |
| Gene 106 | gene | 77,246 | 77,584 | 339 | reverse |
| ATP-dependent protease subunit CDS | CDS | 77,585 | 78,145 | 561 | reverse |
| Gene 107 | gene | 77,585 | 78,145 | 561 | reverse |

| BLAST genes | Description | Max score | Total score | Query cover | E-value | Identity | Accession |
| --- | --- | --- | --- | --- | --- | --- | --- |
| Gene 1 | Pseudoalteromonas phage pYD6-A genomic sequence | 392 | 392 | 95% | 7.00E-105 | 73% | JF974296.1 |
| Gene 4 | Vibrio phage VBP32 genomic sequence | 1323 | 1323 | 98% | 0 | 79% | HQ634196.1 |
| Gene 8 | Pseudoalteromonas phage pYD6-A genomic sequence | 168 | 168 | 48% | 1.00E-37 | 71% | JF974296.1 |
| Gene 10 | Vibrio phage henriette 12B8, complete genome | 170 | 170 | 100% | 3.00E-38 | 70% | HQ316582.1 |
| Gene 11 | Pseudoalteromonas phage pYD6-A genomic sequence | 84.2 | 84.2 | 16% | 1.00E-11 | 67% | JF974296.1 |
| Gene 14 | Pseudoalteromonas phage pYD6-A genomic sequence | 105 | 105 | 52% | 7.00E-19 | 72% | JF974296.1 |
| Gene 15 | Pseudoalteromonas phage pYD6-A genomic sequence | 475 | 475 | 99% | 8.00E-130 | 74% | JF974296.1 |
| Gene 16 | Vibrio phage VBP32 genomic sequence | 1000 | 1000 | 92% | 0 | 72% | HQ634196.1 |
| Gene 17 | Pseudoalteromonas phage pYD6-A genomic sequence | 86 | 86 | 100% | 6.00E-13 | 66% | JF974296.1 |
| Gene 18 | Pseudoalteromonas phage pYD6-A genomic sequence | 408 | 408 | 82% | 1.00E-109 | 70% | JF974296.1 |
| Gene 19 | Pseudoalteromonas phage pYD6-A genomic sequence | 1384 | 1384 | 100% | 0 | 81% | JF974296.1 |
| Gene 20 | Pseudoalteromonas phage pYD6-A genomic sequence | 246 | 246 | 93% | 5.00E-61 | 71% | JF974296.1 |
| Gene 21 | Vibrio phage VBP32 genomic sequence | 159 | 247 | 55% | 8.00E-35 | 74% | HQ634196.1 |
| Gene 26 | Pseudoalteromonas phage pYD6-A genomic sequence | 78.8 | 78.8 | 44% | 1.00E-10 | 71% | JF974296.1 |
| Gene 28 | Vibrio phage VBP32 genomic sequence | 277 | 277 | 82% | 4.00E-70 | 71% | HQ634196.1 |
| Gene 29 | Vibrio phage VBP32 genomic sequence | 300 | 300 | 88% | 3.00E-77 | 71% | HQ634196.1 |
| Gene 30 | Pseudoalteromonas phage pYD6-A genomic sequence | 762 | 762 | 93% | 0 | 69% | JF974296.1 |
| Gene 32 | Vibrio phage VBP32 genomic sequence | 138 | 190 | 63% | 3.00E-28 | 66% | HQ634196.1 |
| Gene 34 | Francisella noatunensis subsp. noatunensis FSC772, complete genome | 143 | 143 | 60% | 8.00E-30 | 66% | CP022207.1 |
| Gene 35 | Francisella noatunensis subsp. noatunensis FSC772, complete genome | 237 | 237 | 37% | 9.00E-58 | 69% | CP022207.1 |
| Gene 37 | Vibrio phage VBP32 genomic sequence | 159 | 159 | 99% | 4.00E-35 | 69% | HQ634196.1 |
| Gene 40 | Vibrio phage VBP32 genomic sequence | 152 | 152 | 29% | 2.00E-32 | 69% | HQ634196.1 |
| Gene 45 | Pseudoalteromonas phage pYD6-A genomic sequence | 452 | 622 | 84% | 3.00E-122 | 67% | JF974296.1 |
| Gene 50 | Vibrio phage VBP32 genomic sequence | 412 | 412 | 91% | 1.00E-110 | 69% | HQ634196.1 |
| Gene 56 | Vibrio phage VBP32 genomic sequence | 165 | 165 | 37% | 2.00E-36 | 72% | HQ634196.1 |
| Gene 63 | Vibrio phage VBP32 genomic sequence | 69.8 | 69.8 | 63% | 3.00E-08 | 72% | HQ634196.1 |
| Gene 64 | - |  |  |  |  |  |  |
| Gene 65 | Vibrio phage VBP32 genomic sequence | 226 | 226 | 85% | 9.00E-55 | 67% | HQ634196.1 |
| Gene 70 | Vibrio phage VBP32 genomic sequence | 80.6 | 80.6 | 40% | 3.00E-11 | 72% | HQ634196.1 |
| Gene 79 | Pseudoalteromonas phage pYD6-A genomic sequence | 120 | 211 | 50% | 1.00E-22 | 67% | JF974296.1 |
| Gene 90 | Vibrio phage VBP32 genomic sequence | 80.6 | 134 | 27% | 8.00E-11 | 73% | HQ634196.1 |
| Gene 97 | Vibrio phage VBP32 genomic sequence | 102 | 102 | 82% | 6.00E-18 | 70% | HQ634196.1 |
| Gene 104 | Vibrio phage VBP47 genomic sequence | 73.4 | 73.4 | 47% | 3.00E-09 | 74% | HQ634194.1 |
| Gene 106 | Vibrio phage VBP32 genomic sequence | 86 | 86 | 88% | 6.00E-13 | 66% | HQ634196.1 |
| Gene 107 | Vibrio phage VBP32 genomic sequence | 471 | 471 | 93% | 7.00E-129 | 80% | HQ634196.1 |
