# Supplementary material for: Isolation and characterization of a N4-like lytic bacteriophage infecting Vibrio splendidus, a pathogen of fish and bivalves
Source: PLoS One. 2017 Dec 28;12(12):e0190083. doi: 10.1371/journal.pone.0190083 (PMC5746245; doi:10.1371/journal.pone.0190083)

Set	Forward primer	Reverse primer	Purpose	Product size (bp)
**1**	CGTGCGTCACCGAGGAGACC	GCGTAGCGCCTACATCAGCA	Circularization	460
**2**	CGTACCTCTGTTGGCAACCC	GTAGCTCCCGGTGGAACCAA	Circularization	373
**Pos1**	GCGCGAAACATAGACTGCTG	GAGAACAACCATGGACACGG	Positive control	255
**Pos2**	GCTTAGCCGCAGTTCATTCG	CTACGGTACGTGCGCCTAGC	Positive control	292

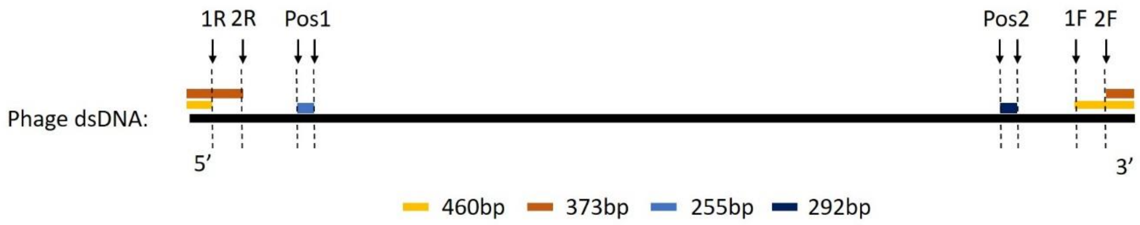

Supplement: S1 Fig — The relative position of the primers as well as the genomic parts expected to be amplified, are also schematically depicted below. (PDF) [file pone.0190083.s002.pdf]
