# Supplementary material for: Isolation and characterization of a N4-like lytic bacteriophage infecting Vibrio splendidus, a pathogen of fish and bivalves
Source: PLoS One. 2017 Dec 28;12(12):e0190083. doi: 10.1371/journal.pone.0190083 (PMC5746245; doi:10.1371/journal.pone.0190083)
Supplement: S2 Fig — (PDF) [file pone.0190083.s003.pdf]

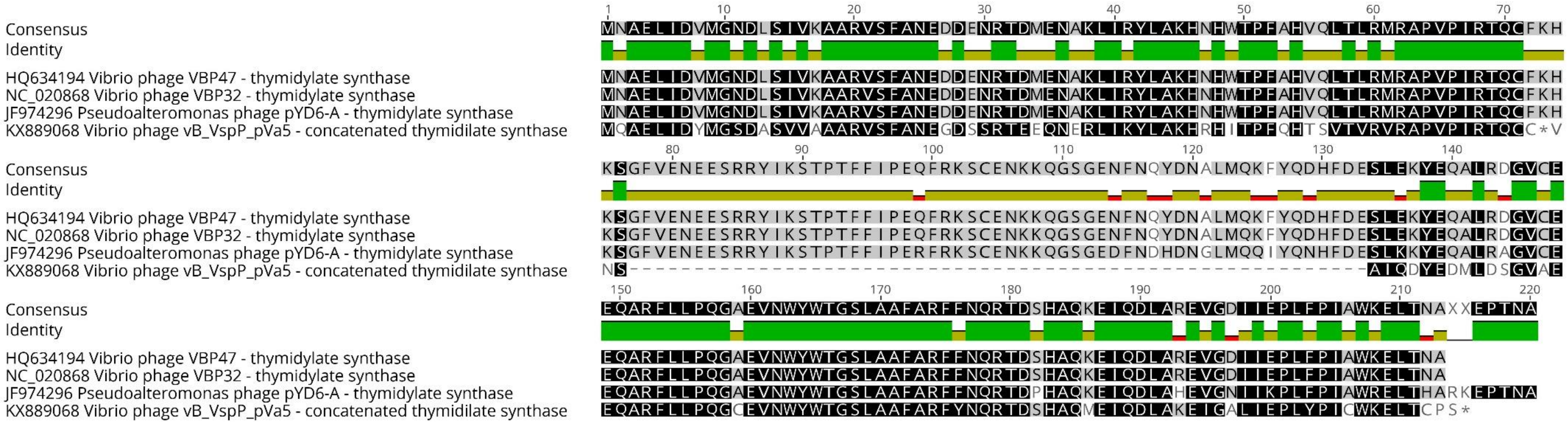
Consensus
Identity
HQ634194 Vibrio phage VBP47 - thymidylate synthase
NC_020868 Vibrio phage VBP32 - thymidylate synthase
JF974296 Pseudoalteromonas phage pYD6-A - thymidylate synthase
KX889068 Vibrio phage vB_VspP_pVa5 - concatenated thymidilate synthase
MNAELIDVMGNDLSIVKAARVSFANEDDENRTDMENAKLIRYLAKHNHWTPFAHVQLTLRMRAPVPIRTQCFKH
MQAELIDYMGSDASVVAAARVSFANEGDSSRTEEQNERLIKYLAKHRHITPFQHTSVTVRVRAPVPIRTQCC*V
KSGFVENEESRRYIKSTPTFFIPEQFRKSCENKKQGSGENFNQYDNALMQKFYQDHFDESLEKYEQALRDGVCE
KSGFVENEESRRYIKSTPTFFIPERFRKSCENKKQGSGEDFNDHDNGLMQQIYQNHFDESLKKYEQALRAGVCE
NS------------------------------------------------------------AIQDYEDMLDSGVAE
EQARFLLPQGAEVNWYWTGSLAAFARFFNQRTDSHAQKEIQDLAREVGDIIEPLFPIAWKELTNAXXEPTNA
EQARFLLPQGAEVNWYWTGSLAAFARFFNQRTDSHAQKEIQDLAREVGDIIEPLFPIAWKELTNA
EQARFLLPQGAEVNWYWTGSLAAFARFFNQRTDPHAQKEIQDLAHEVGNIIKPLFPIAWRELTHARKEPTNA
EQARFLLPQGCEVNWYWTGSLAAFARFYNQRTDSHAQMEIQDLAKEIGALIEPLYPICWKELTCPS*
